# Erratum for “Echocardiographic values for normal conditioned and unconditioned North American whippets”

**DOI:** 10.1111/jvim.16835

**Published:** 2023-08-22

Stepien RL, Kellihan HB, Visser LC, Wenholz L, Luis Fuentes V. Echocardiographic values for normal conditioned and unconditioned North American whippets. J Vet Intern Med. 2023;37(3):844‐855. doi:10.1111/jvim.16691

In Table 4, decimal points have been added to two items under “Structural measures” in the first column.

LVIDdN (cm/kg^332^) should read LVIDdN (cm/kg^.332^)

LVIDsN (cm/kg^433^) should read LVIDsN (cm/kg^.433^)

The authors apologize for the error.

